# Supplementary material for: Interpretable deep learning for the remote characterisation of ambulation in multiple sclerosis using smartphones
Source: Sci Rep. 2021 Jul 12;11:14301. doi: 10.1038/s41598-021-92776-x (PMC8275610; doi:10.1038/s41598-021-92776-x)
Supplement: Supplementary file 1 — Supplementary figures. [file 41598_2021_92776_MOESM1_ESM.pdf]

INTERPRETABLE DEEP LEARNING FOR THE REMOTE CHARACTERISATION OF AMBULATION IN MULTIPLE SCLEROSIS USING SMARTPHONES

A PREPRINT

Andrew P. Creagh*
Institute of Biomedical Engineering
University of Oxford, UK
andrew.creagh@eng.ox.ac.uk

Florian Lipsmeier
Roche Innovation Center
Basel, CH
florian.lipsmeier@roche.com

Michael Lindemann†
Roche Innovation Center
Basel, CH
michael.lindemann@roche.com

Maarten De Vos†
Department of Electrical Engineering
KU Leuven, BE
maarten.devos@kuleuven.be

Supplementary Material

1 Datasets

1.1 UCI HAR

The "Human Activity Recognition (HAR) Using Smartphones Data Set", denoted UCI (*smartphone*) HAR, is a publicly available dataset for HAR [1]. The labelled dataset consists of 10299 instances of labelled activities (walking, stairs, sitting, standing and laying). Measurements were collected from 30 individuals ranging in age from 19 to 48 years recorded wearing a smartphone (Samsung Galaxy SII, sampled at 50 Hz) affixed to the waist. The UCI HAR dataset can be found at: `https://archive.ics.uci.edu/ml/datasets/human+activity+recognition+using+smartphones`

1.2 WISDM

The "WISDM (Wireless Sensor Data Mining) Smartphone and Smartwatch Activity and Biometrics Dataset" is a publicly available dataset for HAR, among other ADL tasks [2] [3]. Data was collected from 51 subjects, ranging in age from 18-25, each of which had a smartphone (Google Nexus 5/5x or Samsung Galaxy S5 sampled at 20 Hz) in their pocket and a smartwatch on their arm, where they performed 18 different tasks for 3 minutes at a time. Those activities of interest for this study were (walking, jogging, stairs, sitting and standing). The WISDM dataset can be found at: `https://archive.ics.uci.edu/ml/datasets/WISDM+Smartphone+and+Smartwatch+Activity+and+Biometrics+Dataset+`

*Corresponding author: andrew.creagh@eng.ox.ac.uk;
†Shared last authorship;

2 Methodology

2.1 Model Architecture

Feature learning was performed by applying a series of one-dimensional kernels on the raw sensor signal with an input (channel 1-4), as per [4]: $\mathbf{X}_n = (\boldsymbol{a}_x, \boldsymbol{a}_y, \boldsymbol{a}_z, \|\boldsymbol{a}\|)^\top$, where $\boldsymbol{a}$ are acceleration vectors for the x-, y- and z- components containing samples $\boldsymbol{a} = (x_1, x_2, ..., x_T)$ and $\|\boldsymbol{a}\|$ refers to original orientation invariant signal magnitude. The DCNN structure in this study adapted the CNN architecture proposed by [5], which consisted of four causal convolutional blocks: the first block (CNN #1) learned 32 filters with a width of 9 samples and stride length of 1 (9×1); CNN #2 and CNN #3 incorporated 64 filters, with width (3×1); CNN #4 learned 128 filters with a with of 6 (6×1). Varying depths of dense fully connected layers were experimented with: n=128, n=64, n=32 nodes at each downstream layer respectively. To guarantee determinism between models, all CNN kernel and DNN layer weights were initialised with a He normal initialiser [6] maintaining the same seeding throughout. This architecture also utilised l_2-norm regularisation ($\lambda = 1e^{-3}$) and batch normalisation (BN) layers [7], which have been proposed in similar networks characterising gait from smartphone inertial sensors [8], [9]. BN normalises the activations of the previous layer at each batch with unit variance and was implemented with momentum for the moving mean and the moving variance ($momentum = 0.99$) and $\epsilon = 1e^{-2}$ to avoid division by zero. Max pooling operations were also applied with pool size p=2 and down-scaled by stride factor s=2, as per [5].

2.2 Model Parametrisation

Models were trained to minimise a cross-entropy loss function:

$$E(\mathbf{w}) = -\sum_{n=1}^{N}\sum_{k=1}^{K} y_{kn} \ln \hat{y}_{kn}(\mathbf{x}_n, \mathbf{w}) \tag{1}$$

where the binary target variables $y_k \in 0, 1$ had a 1-of-K coding scheme indicating the class k, depending on task (i.e. the number of classes) analysed. The network outputs were predicted by a softmax function. The network outputs are are thus interpreted as $\hat{y}_k(\mathbf{x}, \mathbf{w}) = p(y_k = 1|\mathbf{x})$, where the output unit activation is defined by a softmax function (where the $\sum_k \hat{y}_k = 1$):

$$\hat{y}_k(\mathbf{x}_n, \mathbf{w}) = \frac{\exp(a_k(\mathbf{x}, \mathbf{w}))}{\sum_j \exp(a_j(\mathbf{x}, \mathbf{w}))} \tag{2}$$

and a_k are each of the network activations before the final softmax layer. As such, $\hat{y}_k$ can be thought of as the probability that the current data matrix $\mathbf{x}$ belongs to class k.

All DCNN models were trained using *Adam* optimization algorithm with ($\beta_1 = 0.9, \beta_2 = 0.999$), the exponential decay rates for the moment estimates of the gradient [10], [11]. This study also experimented with varying initial learning rates ($lr = \{1\text{e}{-2}, 5\text{e}{-2}, 1\text{e}{-3}, \ldots, 5\text{e}{-5}, 1\text{e}{-5}\}$) and minibatch size ($bs = \{64, 128, 250, 500\}$). For the fine-tuning process, the use of lower learning rates have shown to work best as the source-learned weights should not deviate too greatly during the fine-tuning optimisation, which could risk losing the transferred information [12], [13]. The architecture and parameters that maximised each classification task can be found in appendix table 1. All models in this work were developed using using the Keras framework 2.2.4 [14] with a Tensorflow 1.14 (Google Inc., California) back-end [15].

3 Supplementary Results

Table 1: **Parameters that maximised each target classification task, $\mathcal{T}_T$.**

$\mathcal{T}_T$	$\mathcal{D}_S$	FC DNN[1]	Learning Rate	Mini-Batch Size
HAR	UCI HAR	$\{64, 32, 5\}$	1e−3	128
	WISDM	$\{5\}$	1e−4	128
HC vs. PwMSmild	(end-to-end)	$\{2\}$	1e−3	64
	(UCI HAR, fixed)	$\{2\}$	1e−3	250
	(WISDM, fixed)	$\{64, 32, 2\}$	5e−3	250
	(UCI HAR→FL)	$\{2\}$	1e−5	250
	(WISDM→FL)	$\{32, 2\}$	1e−5	250
PwMSmild vs. PwMSmod	(end-to-end)	$\{32, 2\}$	1e−3	64
	(UCI HAR, fixed)	$\{2\}$	1e−2	250
	(WISDM, fixed)	$\{32, 2\}$	1e−3	250
	(UCI HAR→FL)	$\{64, 32, 2\}$	1e−4	250
	(WISDM→FL)	$\{64, 32, 2\}$	5e−5	250
HC vs. PwMSmod	(end-to-end)	$\{32, 2\}$	1e−2	250
	(UCI HAR, fixed)	$\{2\}$	1e−2	128
	(WISDM, fixed)	$\{2\}$	1e−3	250
	(UCI HAR→FL)	$\{32, 2\}$	5e−5	250
	(WISDM→FL)	$\{64, 32, 2\}$	5e−5	250
HC vs. PwMSmild vs. PwMSmod	(end-to-end)	$\{3\}$	1e−4	64
	(UCI HAR→FL)	$\{3\}$	1e−5	250
	(WISDM→FL)	$\{3\}$	1e−5	250

[1] Neural network layers and the number of hidden layers at each node, including final softmax output.

(a) **PwMSmild predicted as HC**

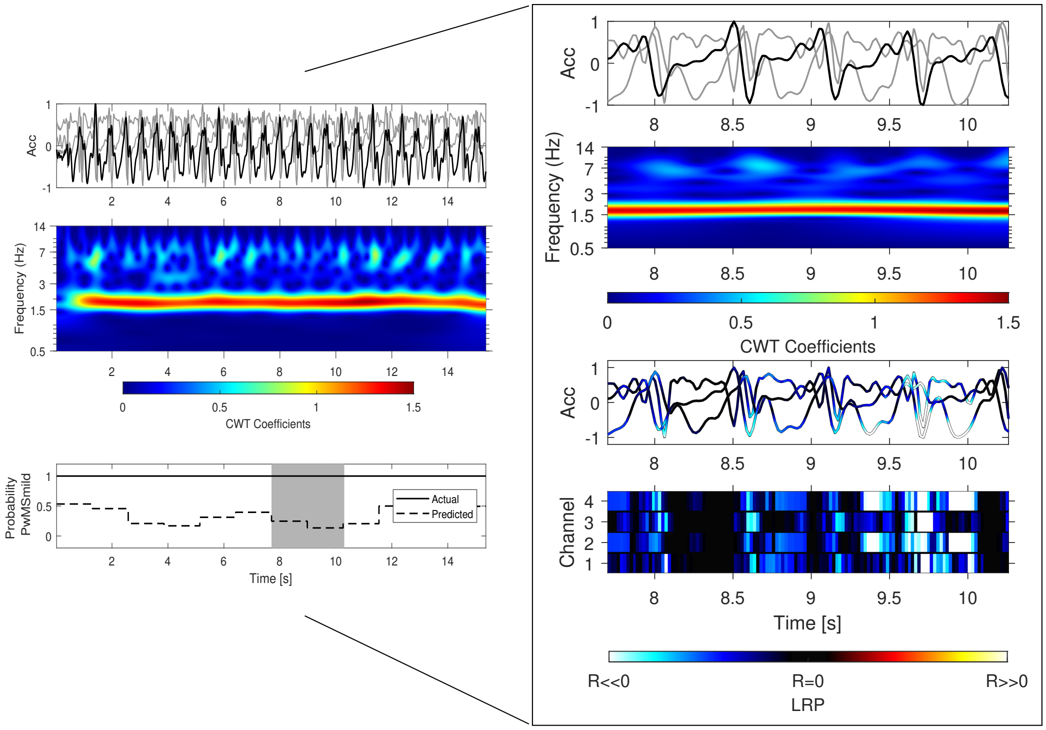

(b) **PwMSmod predicted as HC**

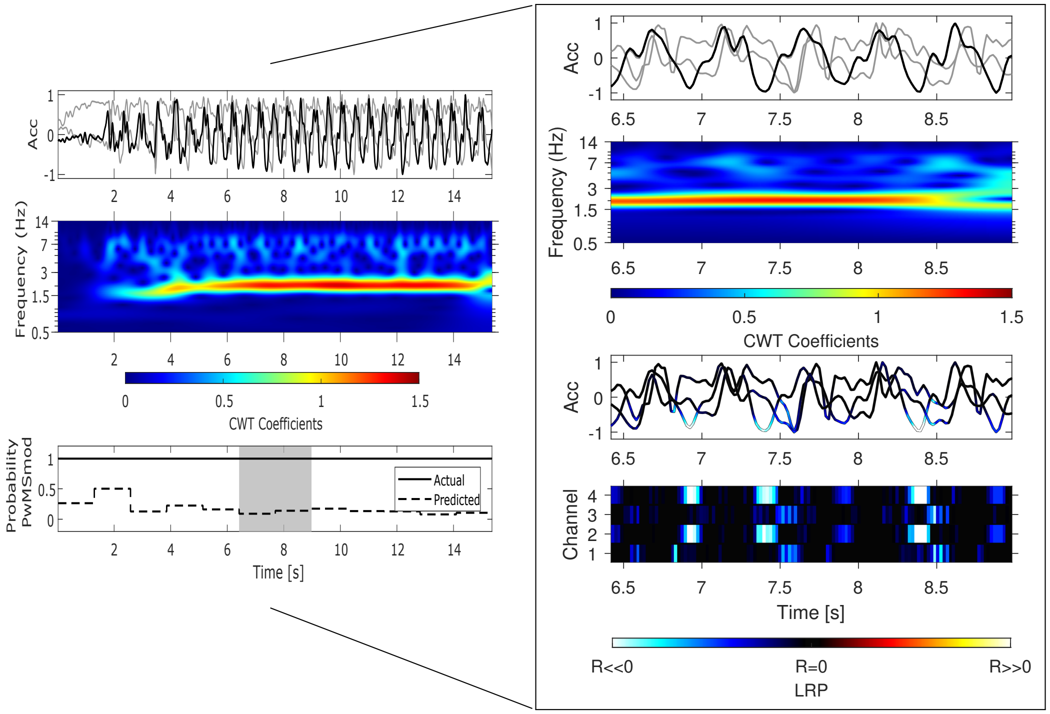

Figure 1: **Panel plot illustrating example performance from a section of misclassified (a) PwMSmild as HC; (b) PwMSmod as HC 2MWT (false negatives) which can be visually interpreted using LRP decomposition and CWT frequency analysis.**

(c) **HC predicted as PwMSmod**

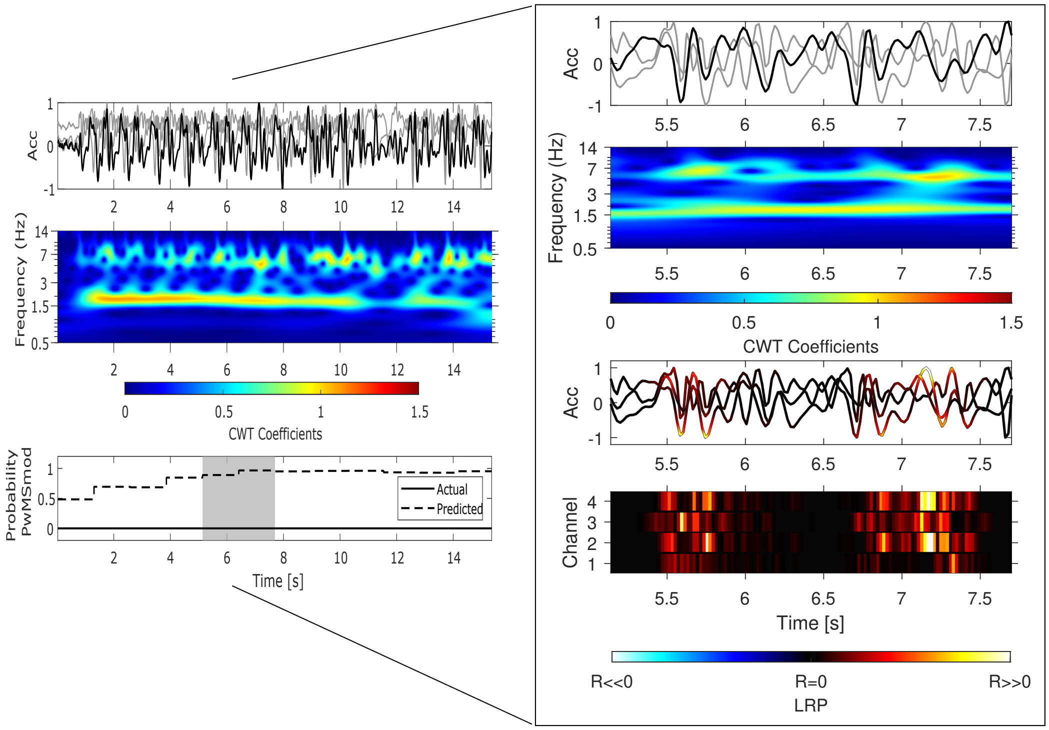

(d) **PwMSmild predicted as PwMSmod**

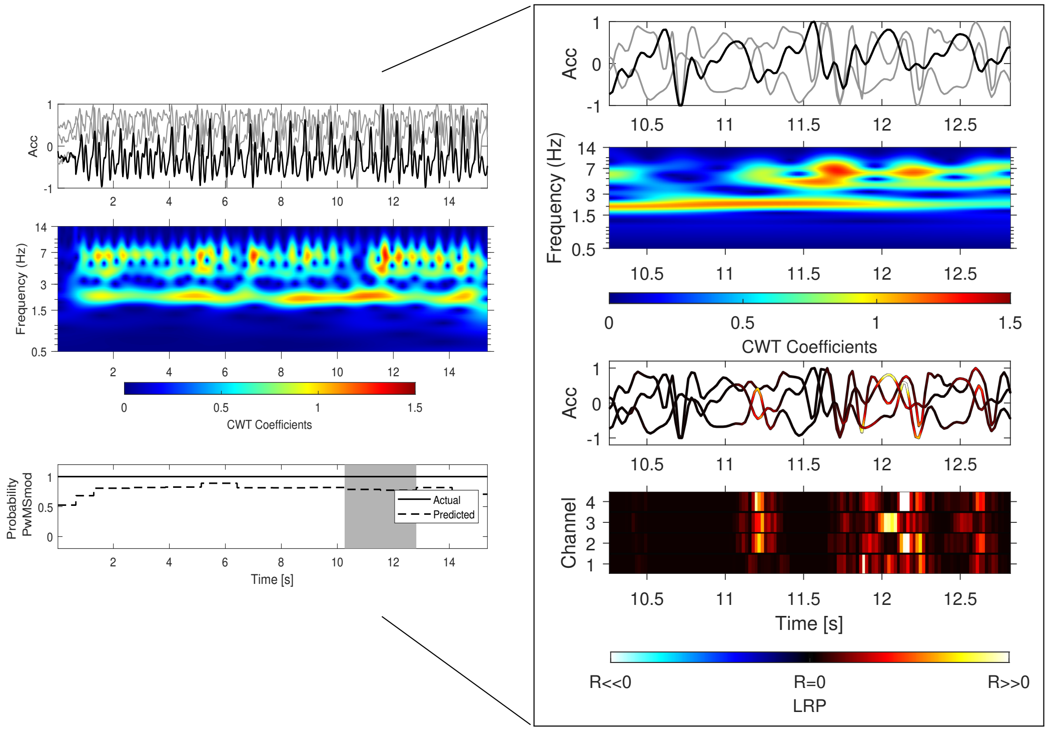

Figure 1: **Cont.: Panel plot illustrating example performance from a section of misclassified (c) HC as PwMSmod; (d) PwMSmild as PwMSmod 2MWT (false positives) which can be visually interpreted using LRP decomposition and CWT frequency analysis.**

References

[1] D. Anguita, A. Ghio, L. Oneto, *et al.*, "A public domain dataset for human activity recognition using smartphones," in *Esann*, 2013.

[2] G. M. Weiss, K. Yoneda, and T. Hayajneh, "Smartphone and smartwatch-based biometrics using activities of daily living," *IEEE Access*, vol. 7, pp. 133 190–133 202, 2019.

[3] G. M. Weiss, J. L. Timko, C. M. Gallagher, *et al.*, "Smartwatch-based activity recognition: A machine learning approach," in *IEEE-EMBS International Conference on Biomedical and Health Informatics (BHI)*, IEEE, 2016, pp. 426–429.

[4] M. Gadaleta and M. Rossi, "Idnet: Smartphone-based gait recognition with convolutional neural networks," *Pattern Recognition*, vol. 74, no. Supplement C, pp. 25–37, 2018.

[5] Q. Zou, Y. Wang, Q. Wang, *et al.*, "Deep learning-based gait recognition using smartphones in the wild," *IEEE Transactions on Information Forensics and Security*, vol. 15, pp. 3197–3212, 2020.

[6] K. He, X. Zhang, S. Ren, and J. Sun, "Delving deep into rectifiers: Surpassing human-level performance on imagenet classification," in *Proceedings of the IEEE international conference on computer vision*, 2015, pp. 1026–1034.

[7] S. Ioffe and C. Szegedy, "Batch normalization: Accelerating deep network training by reducing internal covariate shift," *arXiv preprint arXiv:1502.03167*, 2015.

[8] J. Prince, F. Andreotti, and M. De Vos, "Multi-source ensemble learning for the remote prediction of parkinson's disease in the presence of source-wise missing data," *IEEE Transactions on Biomedical Engineering*, vol. 66, no. 5, pp. 1402–1411, 2018.

[9] H. Zhang, K. Deng, H. Li, *et al.*, "Deep learning identifies digital biomarkers for self-reported parkinson's disease," *Patterns*, p. 100 042, 2020.

[10] D. P. Kingma and J. Ba, "Adam: A method for stochastic optimization," *arXiv preprint arXiv:1412.6980*, 2014.

[11] I. Goodfellow, Y. Bengio, and A. Courville, *Deep learning*. MIT press, 2016.

[12] M. Oquab, L. Bottou, I. Laptev, and J. Sivic, "Learning and transferring mid-level image representations using convolutional neural networks," in *Proceedings of the IEEE conference on computer vision and pattern recognition*, 2014, pp. 1717–1724.

[13] M. T. Martinez and P. D. Leon, "Falls risk classification of older adults using deep neural networks and transfer learning," *IEEE Journal of Biomedical and Health Informatics*, pp. 1–1, 2019.

[14] F. Chollet, *Deep Learning mit Python und Keras: Das Praxis-Handbuch vom Entwickler der Keras-Bibliothek*. MITP-Verlags GmbH & Co. KG, 2018.

[15] M. Abadi, A. Agarwal, P. Barham, *et al.*, "Tensorflow: Large-scale machine learning on heterogeneous distributed systems," *arXiv preprint arXiv:1603.04467*, 2016.
